# DNA Repair and Photoprotection: Mechanisms of Overcoming Environmental Ultraviolet Radiation Exposure in Halophilic Archaea

**DOI:** 10.3389/fmicb.2017.01882

**Published:** 2017-09-29

**Authors:** Daniel L. Jones, Bonnie K. Baxter

**Affiliations:** Department of Biology, Great Salt Lake Institute, Westminster College, Salt Lake City, UT, United States

**Keywords:** halophilic archaea, ultraviolet radiation, DNA damage, DNA repair, photoprotection

## Abstract

Halophilic archaea push the limits of life at several extremes. In particular, they are noted for their biochemical strategies in dealing with osmotic stress, low water activity and cycles of desiccation in their hypersaline environments. Another feature common to their habitats is intense ultraviolet (UV) radiation, which is a challenge that microorganisms must overcome. The consequences of high UV exposure include DNA lesions arising directly from bond rearrangement of adjacent bipyrimidines, or indirectly from oxidative damage, which may ultimately result in mutation and cell death. As such, these microorganisms have evolved a number of strategies to navigate the threat of DNA damage, which we differentiate into two categories: DNA repair and photoprotection. Photoprotection encompasses damage avoidance strategies that serve as a “first line of defense,” and in halophilic archaea include pigmentation by carotenoids, mechanisms of oxidative damage avoidance, polyploidy, and genomic signatures that make DNA less susceptible to photodamage. Photolesions that do arise are addressed by a number of DNA repair mechanisms that halophilic archaea efficiently utilize, which include photoreactivation, nucleotide excision repair, base excision repair, and homologous recombination. This review seeks to place DNA damage, repair, and photoprotection in the context of halophilic archaea and the solar radiation of their hypersaline environments. We also provide new insight into the breadth of strategies and how they may work together to produce remarkable UV-resistance for these microorganisms.

## Saline Systems and Ultraviolet (UV) Light

Halophilic archaea are the predominant residents of hypersaline extreme environments, taxonomically classified within the family *Halobacteriaceae*, order *Halobacteriales*. Most require high salinity for survival or growth (from 2 M to upward of 5 M NaCl at saturation) and lyse in water that is lower in ionic strength (Oren, 1994). Remarkably, they can live in the salt-saturated fluid inclusions of salt crystals (e.g., Fendrihan et al., 2009). The salt lakes, ponds, and deposits inhabited by these microorganisms present challenges in addition to high salinity, one being high exposure to solar UV radiation (that which reaches Earth is divided by wavelength range into UV-A, 315 to 400 nm, and UV-B, 280 to 315 nm). Does the salt in the brine environment impact the exposure of halophilic archaea to UV-induced DNA damage by increasing light penetration? It is clear that at least UV-A radiation penetrates more deeply in saline water (Huovinen et al., 2003). Others have noted that areas of high dissolved organic carbon (DOC) can attenuate UV light (Hammer and Haynes, 1978; Arts et al., 2000), lessening its penetration, but wind activity and shallow waters, typical in salt lakes, increase UV penetration in the high DOC areas as well (Arts et al., 2000).

**FIGURE 1:**
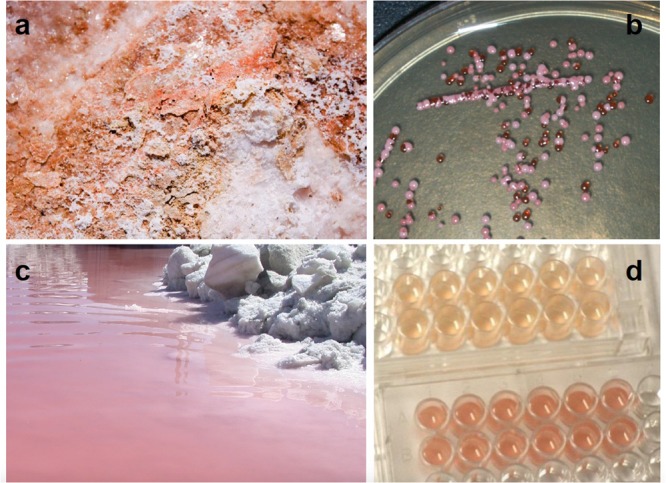
Carotenoid pigmentation in Great Salt Lake (Utah, United States) halophilic archaea **(a)** embedded in a shoreline salt crust, **(b)** growing in colonies on salt agar, and **(c)** coloring the north arm water pink. **(d)** A Great Salt Lake *Halorubrum* species was grown in the absence (top) and presence (bottom) of full spectrum light, demonstrating the impact of light on carotenogenesis (Baxter et al., 2007).

Some salt lakes, such as Great Salt Lake, are high in altitude and thus, have increased UV exposure. Depending on the wavelength of UV light measured, the increase of UV exposure (300–370 nm) ranges between 9 and 24% per one thousand meters (Blumthaler et al., 1997). Also, salt in and around such lakes causes mobilization of atmospheric chlorine, which has depleted ozone concentrations, leading to more UV exposure (Stutz et al., 2002). Therefore, halophilic archaea may experience a significant dose of UV light in their native environments. However, halophilic archaea in desiccated salty shores or evaporite formations (**Figure 1a**) may receive less UV exposure. In the lab, such microorganisms inhabiting salt crystal fluid inclusions received some protection from ultraviolet light radiation (Fendrihan et al., 2009), even while the salt allows the transmission of visible light (Rothschild, 1990; Cockell and Raven, 2004).

While not photosynthetic, halophilic archaea are facultative phototrophic organisms (Bryant and Frigaard, 2006), and their growth is enhanced when cultured in the light (Oren, 1994). Some species possess light-driven proton pumps, bacteriorhodopsins, that can drive ATP synthesis (e.g., Blaurock and Stoeckenius, 1971; DasSarma et al., 2001; Lanyi, 2004), which are not necessary for survival, but do contribute free energy. Halophilic archaea may have more than one rhodopsin; for example, *Haloarcula marismortui* has six homologous rhodopsin genes (Baliga et al., 2004), and *Halobacterium salinarum* (e.g., strain NRC-1) uses two distinct sensory rhodopsins to accomplish color-sensitive phototaxis (Lanyi, 2004). The energetic benefits (ATP synthesis) of phototropism necessitate routine exposure to sunlight, resulting in high levels of UV radiation. Exposure to visible light also regulates genes for the formation of gas vesicles (Englert et al., 1992; Walsby, 1994; Pfeifer, 2012), which, along with flagella, allow halophilic archaea to move up in the water column toward sunlight.

Excessive exposure to sunlight in their environment has likely contributed to the evolution of other photobiology for halophilic archaea. For example, these microorganisms display remarkable UV resistance, first noted by Dundas and Larsen (1963). This observation is well-supported by more recent studies; for example, Shahmohammadi et al. (1997) observed a D_37_ value (the UV-radiation dose corresponding to 37% survival) for *H. salinarum* 21.2 times higher than that of *Escherichia coli*; Martin et al. (2000) and Baxter et al. (2007) also noted a nearly 10-fold increase in UV resistance of a Great Salt Lake *Halorubrum* isolate when compared with *E. coli.* Moreover, *Halobacterium* species can endure a UV dose of between 39 and 110 J/m^2^ with no impact on viability (Martin et al., 2000; Baliga et al., 2004). Clearly, halophilic archaea have strategies for surviving and thriving in high UV radiation despite the threats of cellular and DNA damage. UV-B, especially, affects both cellular proteins and DNA since these molecules absorb in this wavelength range; however, this review will focus only on DNA.

Halophilic archaea live in high salinity environments with excessive UV exposure and desiccating conditions. Herein, we explain the secrets of their success in navigating DNA damage with both photoprotective mechanisms, which serve as a “first line of defense,” and DNA repair.

## UV-Induced DNA Damage

**FIGURE 2:**
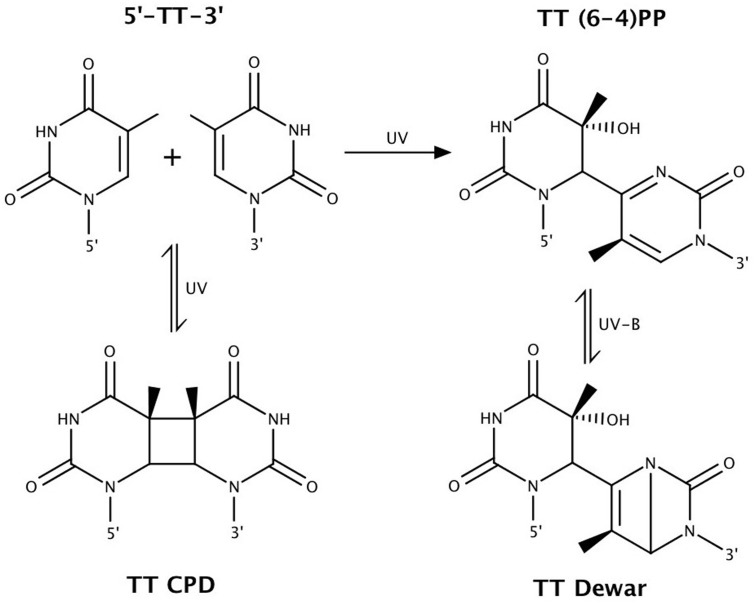
Bipyrimidine lesions, the primary form of ultraviolet (UV)-induced DNA damage. Shown above are TT photolesions. Similar chemistry occurs at the other bipyrimidine sites, with the exception that 5′-CT-3′ sequences only form CPDs (Sinha and Häder, 2002). Figure adapted from Rastogi et al. (2010).

The damaging effects of UV light exposure result in helix-distorting damage to the DNA. This occurs most notably through the induction of cyclobutane pyrimidine dimers (CPDs), pyrimidine (6-4) pyrimidone photoproducts [(6-4)PPs], and the (6-4)PP-related Dewar valence isomers (**Figure 2**) (Yoon et al., 2000; Cadet et al., 2001, 2005; Sinha and Häder, 2002; Friedberg, 2003). Indeed, Moeller et al. (2010) found that these account for approximately 80% of UV-induced photolesions in the halophilic archaeon *Natronomonas pharaonis*. *H. salinarum* and *Haloferax volcanii* were shown to accumulate both CPDs and (6-4)PPs at the same rates as other organisms (McCready, 1996).

Cyclobutane pyrimidine dimers and (6-4)PPs may form between adjacent pyrimidine bases (5′ to 3′: TT, TC, CT, and CC) upon exposure to UV radiation, with the exception that (6-4)PPs do not form at 5′-CT-3′ sequences (Sinha and Häder, 2002). Dewar valence isomers form through a UV-B-induced photoisomerization of (6-4)PPs (Mitchell and Rosenstein, 1987; Matsunaga et al., 1993). CPDs are the predominating photoproduct (Besaratinia et al., 2011). It is estimated that the ratio of CPDs to (6-4)PPs induced by solar radiation is approximately 3:1 (Sinha and Häder, 2002). This ratio is dependent on wavelength, with CPD and (6-4)PP formation more associated with UV-B and UV-C, respectively (Cadet et al., 2005; Besaratinia et al., 2011). Flanking sequences are also implicated in influencing CPD vs. (6-4)PP formation (Mitchell et al., 1991; Yoon et al., 2000). Perdiz et al. (2000) measured in mammalian cells the ratio of CPDs : (6-4)PPs : Dewar isomers to be 1:0.25:0 under 254 nm UV-C, 1:0.12:0.014 under broadband UV-B, and 1:0.18:0.06 under simulated sunlight. These observations demonstrate the importance of using UV-B, rather than UV-C, for studies of solar DNA damage and mutagenesis, an issue raised by Boubriak et al. (2008).

**FIGURE 3:**
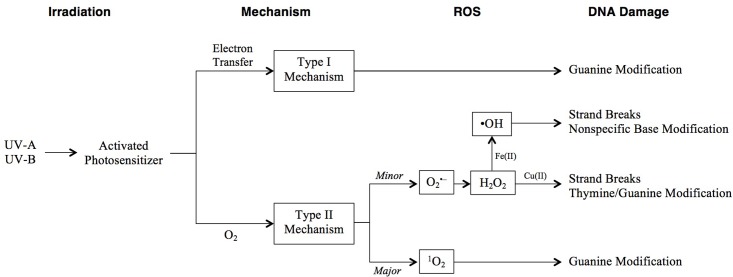
Pathways of photooxidative DNA damage following UV irradiation. DNA damage can occur through two mechanisms: type I involves electron transfer from an excited photosensitizer to a DNA base, while type II is a direct reaction with O_2_ that forms ROS. Resulting specific DNA damage is shown in the final column.

Ultraviolet-A, constituting approximately 95% of solar UV radiation (IARC, 2012), is poorly absorbed by DNA and is associated with DNA damage resulting from the generation of reactive oxygen species (ROS) (**Figure 3**) (Cadet et al., 2001; Kawanishi and Hiraku, 2001; Cadet et al., 2005). These may be produced by absorption of UV-A (or UV-B) photons by, and subsequent activation of, endogenous photosensitizers such as porphyrins and flavins. While the primary focus of the present review is damage to DNA, it should be noted that ROS-induced cell death in *H. salinarum* appears to be more a result of major metabolic interference than DNA lesions (Robinson, 2009), and other biological molecules such as proteins are certainly impacted by UV light exposure (Fendrihan et al., 2009).

Photooxidative DNA damage includes base modifications and strand breaks and occurs through one of two mechanisms (**Figure 3**). The type I mechanism proceeds via electron transfer from an excited photosensitizer to a DNA base, most commonly guanine, as it has the lowest oxidation potential (G < A < T, C < 2-deoxyribose) (Cadet et al., 2001). The type II major mechanism induces guanine modification, and is mediated by singlet oxygen (^1^O_2_) generated by an energy transfer from an excited photosensitizer to molecular oxygen (Kawanishi and Hiraku, 2001). The type II minor mechanism involves the formation of superoxide anion (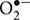), H_2_O_2_, and hydroxyl radicals (•OH). Of these, •OH is the most toxic, causing non-specific base modification and strand breaks (Kawanishi and Hiraku, 2001; Imlay, 2003; Imlay, 2008). It is produced from H_2_O_2_ via the Fe(II)-dependent Fenton reaction. Thus, •OH damage is especially prevalent at Fe(II)-rich DNA sites (Henle et al., 1999). 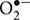 indirectly damages DNA by generating free Fe(II) (Keyer and Imlay, 1996) and by dismutation to H_2_O_2_ (Kawanishi and Hiraku, 2001). H_2_O_2_, in addition to generating •OH, may cause strand breaks or thymine/guanine modification in the presence of Cu(II), although it is the least toxic of the aforementioned ROS.

The consequence of DNA lesions, for any organism, is ultimately mutation or even cell death. When the helix undergoes DNA replication, damaged bases may result in mispairing or replication blocks, leading to mutation or partially replicated genomes (reviewed in Friedberg, 2003). The impact of UV-induced DNA damage on the mutation rate is moderated by photoprotective mechanisms that prevent damage, and perhaps most importantly, DNA repair processes that fix it. Halophilic archaea use both of these strategies, which are explored below.

## DNA Repair of UV-Induced Damage in Halophilic Archaea

DNA repair processes that fix DNA damage are highly conserved in evolution (Eisen and Hanawalt, 1999). Halophilic archaea have robust and efficient systems for repairing different types of damage (reviewed in Kish and DiRuggiero, 2012) and possess genes that share lineages with both eukaryotic cells (e.g., Yeast *rad* genes) and bacteria (*uvr* genes) (DasSarma et al., 2001).

Baliga and others used a systems approach to identify repair systems in the lab model, *H. salinarum* (strain NRC-1), utilizing a combination of gene knockouts, biochemistry assays, comparative genomics and mRNA transcript analyses (2004). This study not only identified genes in dark and light (see below) DNA repair pathways, but also discovered several enzymes involved in oxidative repair. Indeed, halophilic archaea appear to have an arsenal of machines that mitigate the DNA damaging effects of UV exposure (**Table 1**).

**Table 1:** DNA repair systems that address UV-induced damage, all of which have been researched in halophilic archaea, ^∗^with the exception of single-strand break repair.

Repair mechanism	UV photodamage repaired
Photoreactivation	Cyclobutane pyrimidine dimers,
	Pyrimidine (6-4) pyrimidone photoproducts
	Dewar isomers
Nucleotide excision repair	Cyclobutane pyrimidine dimers
(Sub-category: transcription-coupled repair)	Pyrimidine (6-4) pyrimidone photoproducts
	Dewar isomers
Base excision repair	Oxidative base lesions
	Single-strand breaks^∗^
Homologous recombination	Double-strand breaks
	Cyclobutane pyrimidine dimers
	Pyrimidine (6-4) pyrimidone photoproducts
	Dewar isomers

### Direct Photoreactivation

Photoreactivation (PHR), also known as “light repair,” is a process that utilizes visible light to reverse UV-induced lesions, either CPDs or (6-4)PPs, by directly rearranging bonds. A photolyase enzyme recognizes a lesion, binds to the site, and from there it is a single-step chemical process that uses blue to near-UV light energy to return the CPD or (6-4)PP to its original state (Sancar, 2000). The catalytic cycle of photolyases rely on a non-covalently bound cofactor, flavin adenine dinucleotide (FAD) (reviewed in Weber, 2005). Both the ground-state redox properties and the excited-state properties of the FAD cofactor are utilized. All photolyases are homologous across bacteria, archaea and eukaryotes, which suggests this mechanism developed early in evolution (Eisen and Hanawalt, 1999).

Photoreactivation genes, *phr1* and *phr2*, that encode photolyase enzymes have been described in several studies on halophilic archaea (DasSarma et al., 2001; McCready and Marcello, 2003; Baliga et al., 2004; Capes et al., 2011) and the PHR process has been observed in some species and described (Hescox and Carlberg, 1972; Martin et al., 2000; McCready and Marcello, 2003). Interestingly, in gene knockout studies of *phr1* and *phr2*, only *phr2* was associated with PHR in *H. salinarum* (Baliga et al., 2004). The *phr2* gene product did not display (6-4)PP repair activity, only efficient CPD repair (McCready and Marcello, 2003). There may also be species-specific regulation (Kish and DiRuggiero, 2012) since UV irradiation induced transcription of the *ph2* gene in *Halococcus hamelinensis* (Leuko et al., 2011) but not *H. salinarum* (Baliga et al., 2004).

The function of *phr1* is unclear. Kanai et al. (1997) suggested that the *phr1* gene encodes a blue light receptor, descended from ancestral photolyase genes, and may function in circadian rhythms. A study on the evolution of photolyase genes also demonstrates that specificity for CPD vs. (6-4)PP lesions can change through time and across species (Eisen and Hanawalt, 1999).

### Nucleotide Excision Repair

Nucleotide excision repair (NER), or “dark repair,” is a universal and highly conserved system that allows cells to excise DNA lesions including CPDs, (6-4)PPs, and other bulky adducts (Sancar, 1996). Its machinery does not require light for the reactions to occur. There are several proteins involved that carry out this multi-step process involving recognition of the DNA damage (e.g., in bacteria, UvrA), single strand cutting on both the 5′ and 3′ sides of the lesion (UvrB and UvrC), and removal of the damaged strand by a helicase (UvrD). A DNA polymerase must then build a new strand complementary to the undamaged one, and finally, ligase seals the phosphodiester backbone. All halophilic archaea examined have the *uvrABCD* genes (Capes et al., 2011), the necessary DNA polymerases (Kish and DiRuggiero, 2012), and the ligases (e.g., Zhao et al., 2006).

Halophilic archaea species may have eukaryotic homolog NER genes as well as the bacterial UvrABCD system, as homologs from both the XP system (mammalian) and Rad system (yeast) have been described in the archaea domain (Eisen and Hanawalt, 1999). For example, *H. salinarum* has *xpf* and the *rad* genes (*rad2*, *rad3*, *rad25*) (DasSarma et al., 2001; Capes et al., 2011). Rouillon and White (2011) postulated that the XPF-like nuclease (which does 5′ cleavage of the damage site in mammals) may be involved in a different repair pathway, and not NER, since the archaeal XPFs studied have a broader specificity than the nuclease of mammalian cells. Despite the observation of eukaryotic repair genes, at least the lab model species *H. salinarum* appears to depend entirely on the UvrABCD system for NER (Crowley et al., 2006), but it is not clear if this is true for all other halophilic archaea. It has been theorized that other genes may be involved in repair-supportive processes such as addressing damage causing stalled replication forks (Boubriak et al., 2008).

An early investigation of *H. salinarum* suggested halophilic archaea do not have NER (Sharma et al., 1984); however, this was later corrected in the literature (McCready, 1996; McCready and Marcello, 2003). To date, a number of halophilic archaea species have been shown to use NER to repair photodamage, including *H. volcanii* (McCready, 1996), *H. salinarum* (McCready, 1996; McCready and Marcello, 2003; Baliga et al., 2004; Boubriak et al., 2008), and a Great Salt Lake *Halorubrum* species (Baxter et al., 2007). Verifying the importance of the UvrABCD system, *H. volcanii* mutants lacking *uvrA* are significantly more UV sensitive than their wild-type counterparts (Lestini et al., 2010). Furthermore, *H. salinarum* mutant studies knocking out the function of UvrA, C, or AC double mutants reduced the repair of CPDs and thus, the survival of these strains (Crowley et al., 2006).

Halophilic archaea are also capable of transcription-coupled repair (TCR), a subpathway of NER that functions in removing RNA-polymerase-arresting DNA lesions from the template strands of active genes (Savery, 2007). Stantial et al. (2016) demonstrated that *H. salinarum* and *H. volcanii* employ TCR to repair CPDs following UV irradiation. A *uvrA* dependence was observed in *H. salinarum*, but not *H. volcanii*. It was proposed that a unique mechanism for TCR exists in halophilic archaea in which NER proteins are recruited by arrested RNA polymerase complexes following lesion recognition by the RNA polymerase itself.

### Base Excision Repair

The base excision repair (BER) pathway removes damaged or modified bases in DNA, which can be caused by UV-induced oxidative damage or other intracellular metabolites that modify the DNA base structure (reviewed in Krokan and Bjørås, 2013). DNA glycosylases that are specific to the particular photooxidative damage cleave the *N*-glycosidic bond between the base and the deoxyribose ring. The DNA backbone is then cleaved by an abasic-site endonuclease and the deoxyribose sugar is removed. The opposite strand provides the template for a repair polymerase to replace the removed nucleotide, and ligase seals the backbone. ROS damage to bases is repaired predominantly by BER across all species studied (Eisen and Hanawalt, 1999; Krokan and Bjørås, 2013) and likely in halophilic archaea as well (Capes et al., 2011).

Base excision repair glycosylase genes include *mutY* (A/G-specific adenine glycosylases), *alkA* (alkyladenine glycosylase), and *nth* (endonuclease III) (Denver et al., 2003; Krokan and Bjørås, 2013). These are found across the halophilic archaea with some exceptions and variations (Capes et al., 2011). Notably, *alkA* is missing from *Haloquadratum walsbyi*, and the *nthA* gene has three variants in some species. Other genes involved in this repair pathway are also present, indicating halophilic archaea have a fully functional BER apparatus. Upon UV-irradiation, Baliga et al. (2004) observed the up-regulation of six genes involved in repair of photooxidative damage.

It is unclear how halophilic archaea handle UV-induced single strand breaks (SSBs). In bacteria, the majority of these are breaks in the backbone and are repaired by ligase, but damage that creates an apurinic or apyrimidinic site is repaired by BER (e.g., Peak and Peak, 1982).

### Homologous Recombination

Homologous recombination (HR) is also employed by cells to repair UV damaged DNA, in particular, double-strand breaks (DSBs), but to a lesser extent, lesions such as CPDs and (6-4)PPs that stall replication forks. Following this damage, there are several steps: DSB recognition, excision at broken ends to create recognition sites, recombinase binding, strand pairing/exchange, branch migration, and branch resolution (Cox, 1991). The RecA protein brings homologous molecules together and facilitates this strand exchange. Recombinational repair can result in mutation as it has the potential to cause genome rearrangements.

In bacteria (e.g., *E. coli*), HR is highly conserved, and there are at least four pathways for the initiation of recombination, all of which produce substrates used by the RecA protein to catalyze the pairing and exchange (Roca and Cox, 1997). Interestingly, despite much focus on NER and BER, HR may play a larger role than generally thought in addressing UV damage. Mutations in the *recA* gene are more sensitive to UV light than NER genes such as *uvrA* (Cox, 1991). The eukaryotic Rad51 family of proteins (e.g., *Saccharomyces cerevisiae*) is related to RecA in bacteria, and homologs are present in at least some species of archaea (Sandler et al., 1996). The archaeal RadA proteins have been shown to function similarly in recombinational repair to RecA/Rad51 (Seitz et al., 1998), and two distinct *radA* genes are found in sequenced halophilic archaea genomes (Capes et al., 2011). Also, halophilic archaea have homologs to the yeast proteins Mre11, an HR nuclease, and Rad50, an HR ATPase, suggesting that the archaeal systems are likely similar in complexity to the eukaryotic yeast model (Woods and Dyall-Smith, 1997).

Halophilic archaea do employ HR following UV assault if DSBs occur. When a *radA* mutant of *H. volcanii* was exposed to UV light, this strain demonstrated sensitivity, which underscores the significance of this repair system for UV damage (Woods and Dyall-Smith, 1997). In wild type *H. salinarum* cells, UV-B or UV-C exposure induced the *radA1* as well as other genes implicated in HR (McCready et al., 2005; Boubriak et al., 2008). Also, in this strain, mutant studies show *mre11* is likely involved in DSB end processing as in eukaryotes, but not *rad50* (Kish and DiRuggiero, 2008), and double mutants of these genes in *H. volcanii* are sensitive to DSB accumulation (Delmas et al., 2009). Halophilic archaea are polyploid (Breuert et al., 2006), and this may create a disadvantage in HR, given that concatemers can form between circular chromosomes as resolution proceeds (Delmas et al., 2009). However, polyploidy may also give the cells more correct sequence templates from which to draw in repairing the damaged area (Kish and DiRuggiero, 2008; Kish and DiRuggiero, 2012).

The HR RecA/Rad51 protein families are also known to induce an “SOS response” to excessive DNA damage, especially when single strands are exposed (Radman, 1975; Janion, 2008). This global response arrests DNA replication and induces genes in repair, mutagenesis and other DNA metabolisms. When looking at UV-induced gene induction in *H. salinarum*, two independent studies noted an increase in *radA1* transcription but not other genes expected for an SOS response (Baliga et al., 2004; Breuert et al., 2006). To date, the SOS response is thought to be absent in halophilic archaea.

## Photoprotection

In addition to their efficient DNA repair, a number of systems have been observed in halophilic archaea that are thought to act as a “first line of defense” from UV light, providing protection from the consequences of habitual exposure to intense UV. These photoprotective systems are thought to prevent damage before it occurs, thereby reducing the impact on, or even photodamage to, the DNA repair machinery.

### Carotenoids

The red-orange and pink colors characteristic of aquatic hypersaline ecosystems such as Great Salt Lake, Utah are attributed to the accumulation of carotenoid pigments within cell membranes of resident halophilic archaea (**Figure 1**). Though not the subject of this review, we should note that there are also halophilic, carotenoid-containing bacteria, such as the *Salinbacter* genus, present in lower abundance.

**FIGURE 4:**
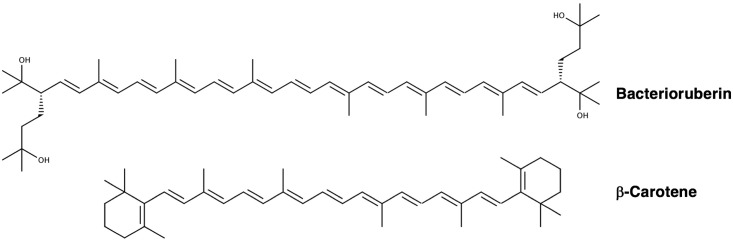
Chemical structures of bacterioruberin and β-carotene (Yang et al., 2015), two major carotenoids produced by halophilic archaea.

These compounds are comprised of long, conjugated hydrocarbon chains that generally possess oxygen-containing functional groups and symmetry about the central carbon (**Figure 4**). Halophilic archaea are distinguished by a unique set of carotenoids (Kelly et al., 1970; Kushwaha et al., 1974, 1975; Marshall et al., 2007), the predominating pigment being bacterioruberin (Kelly et al., 1970; Ronnekleiv, 1995; Lobasso et al., 2008; Mandelli et al., 2012; Jehlicka et al., 2013; Naziri et al., 2014; Yatsunami et al., 2014), a compound implicated in protecting from UV photodamage (Shahmohammadi et al., 1998; Asgarani et al., 1999).

The pathway of carotenoid biosynthesis in halophilic archaea (reviewed in Rodrigo-Baños et al., 2015) begins with the isoprenoid precursor, isopentenyl pyrophosphate, which is converted to geranylgeranyl pyrophosphate, the first carotenoid of the pathway. Two of these molecules are joined to form phytoene, which is subsequently converted to lycopene through stepwise desaturation (Kushwaha et al., 1976). Lycopene gives rise to two of the major carotenoids of halophilic archaea, bacterioruberin and β-carotene. β-carotene is a precursor to retinal. In *H. salinarum*, retinal is incorporated as a chromophore into bacteriorhodopsin, or “purple membrane” protein, which pumps protons out of the cell upon exposure to light (Oesterhelt and Stoeckenius, 1971) to power ATP synthase enzymes. Other retinal-containing, light-energy transducing proteins are found in *H. salinarum*, such as halorhodopsin (Mukohata et al., 1980; Mukohata and Kaji, 1981), sensory rhodopsin, and photorhodopsin (Mukohata et al., 1999).

Carotenoid biosynthesis in halophilic archaea is regulated by a variety of factors including salinity (D’Souza et al., 1997; Lobasso et al., 2008; Biswas et al., 2016), pH (Hamidi et al., 2014; Rodrigo-Baños et al., 2015), oxygen tension (Sumper et al., 1976; Ng et al., 2000; DasSarma et al., 2001), and, of note, light exposure. The pigmentation levels of halophilic archaea grown under bright light are visibly higher than those cultured in the dark (**Figure 1d**) (Baxter et al., 2007). A number of genes connected to the carotenoid biosynthetic pathway that are regulated in response to light (and O_2_) have been identified in *H. salinarum* (reviewed in Ng et al., 2000; DasSarma et al., 2001). Several are organized in the purple membrane regulon (*crtB1*, *blp*, *bat*, *brp*, and *bop*). It has been shown that bacterioruberin synthesis, specifically, the *bop* gene cluster of this species, is induced by low oxygen tension and high light intensity (Shand and Betlach, 1991). It has also been shown that the conversion of lycopene to bacterioruberin (Dundas and Larsen, 1963; Shahmohammadi et al., 1998), as well as β-carotene to retinal (El-Sayed et al., 2002), are enhanced by light in *H. salinarum*. This underscores the physiology of halophilic archaea, which must rise to the surface of the water to utilize their proton pump, but in doing so may encounter photodamage.

How do carotenoids protect halophilic archaea from photodamage? The best-established mechanism is through their antioxidant activity, which prevents photooxidative damage through ROS scavenging (most notably, ^1^O_2_ and •OH quenching) and deactivating excited photosensitizers (Krinsky, 1979; Truscott, 1990; Miller et al., 1996; Saito et al., 1997; Young and Lowe, 2001; Stahl and Sies, 2003; Mandelli et al., 2012; Igielska-Kalwat et al., 2015; Islamian and Mehrali, 2015). The antioxidant capacity of carotenoids increases with the number of conjugated π-bonds as well as the length of the carbon chain. For example, the increased conjugation of bacterioruberin (13 π-bonds) by comparison to β-carotene (9 π-bonds) (**Figure 4**) affords it a higher efficacy of ROS scavenging (Saito et al., 1997). The mechanisms by which carotenoids prevent oxidative damage take place in a manner that leaves them intact (Stahl and Sies, 2003). ^1^O_2_ quenching takes place through a direct transfer of energy between molecules, after which the energy gained by the carotenoid dissipates into the solvent as heat. The quenching of free radicals leads to subsequent reactions; •OH scavenging in particular is thought to play an important role in preventing oxidative damage to membranes (Sies and Stahl, 1995).

Carotenoids then certainly provide antioxidant protection from photochemical damage not only to DNA, but also to membranes and other cell components. This notion is well demonstrated by the increased sensitivity of colorless mutant halophilic archaea to UV irradiation (Dundas and Larsen, 1963; Rodriguez-Valera et al., 1982; Shahmohammadi et al., 1998; Baxter et al., 2007). Dundas and Larsen (1963) were the first to demonstrate that non-pigmented *H. salinarum* cells are sensitive to the damaging effects of light when compared with pigmented cells, despite both cell types growing equally well with no light exposure. The consequence of pigment loss was described as extensive lysis of the irradiated cells. Rodriguez-Valera et al. (1982) also observed membrane lysis of colorless or pale halophilic archaea exposed to intense light. These findings point to the most significant ramifications of intense photooxidative damage occurring outside of DNA.

Carotenoids apparently offer protection from direct forms of DNA photodamage. The formation of CPDs is suppressed by the presence of carotenoids; Baxter et al. (2007) demonstrated that the relative levels of TT damage were decreased 3.5-fold in UV-irradiated *Halorubrum* cells that were rich in pigmentation due to full-spectrum light exposure, when compared to irradiated cells that had been grown in the dark and had reduced carotenoid pigmentation (**Figure 1d**). These findings are in agreement with *in vitro* studies of Asgarani et al. (1999), which demonstrate the formation of CPDs in plasmid DNA is reduced in the presence of bacterioruberin. The specific mechanism through which this form of photoprotection occurs remains unknown (see conclusion). Many studies do suggest direct absorption of UV (e.g., Shahmohammadi et al., 1998). However, carotenoid compounds absorb light in the range of 340–550 nm (Takaichi and Shimada, 1992), whereas the UV spectrum ranges from 200 to 400 nm. Most likely then, they do not afford photoprotection by acting as a complete optical filter (Cockell and Knowland, 1999).

Carotenoids also exhibit interplay with the PHR system. Sharma et al. (1984) examined the UV sensitivity of several pigmented and colorless strains of *Halobacteria* and saw the levels of photoreactivation were reduced in the colorless mutants. The authors suggested the interpretation that the pigments do not play a role in direct absorption of UV, but instead function by supplying energy to photolyase during repair of pyrimidine dimers. However, this does not explain the observation that carotenoids provide photoprotection from UV under photolyase-inhibiting (dark) conditions (Baxter and Zalar, in press). Interestingly, Shahmohammadi et al. (1998) noted the effects of bacterioruberin were more protective in the case of UV exposure in *H. salinarum* than when cells were exposed to ionizing radiation or H_2_O_2_. They suggest the same explanations offered above: absorbance of UV energy by the carotenoid and a supplying of energy to the photoreactivation system. Nevertheless, these explanations do not complete the picture of how carotenoids shield DNA from UV light, particularly in the absence of visible light.

### Oxidative Damage Avoidance

In addition to carotenoids, a number of overlapping pathways for avoiding oxidative damage via ROS detoxification are seen in archaea (reviewed in Pedone et al., 2004). Of particular relevance to the present review are hydroperoxidases and superoxide dismutases. These enzymes work together to prevent oxidative damage through ROS scavenging (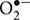 and H_2_O_2_ in particular), and are found widely among aerobic and facultatively anaerobic organisms.

Hydroperoxidases are heme proteins that facilitate the elimination of H_2_O_2_ (Pedone et al., 2004). They are divided into two classes, catalases, which catalyze the decomposition of H_2_O_2_ into O_2_ and H_2_O, and peroxidases, which catalyze the oxidation of other organic compounds by H_2_O_2_. Active catalase and peroxidase enzymes have been reported for *H. salinarum* (Fukumori et al., 1985; Brown-Peterson and Salin, 1995). Bifunctional catalase-peroxidase enzymes have also been observed. That of *H. salinarum* was found to shift between catalase- and peroxidase-dominant activity in response to pH and NaCl concentration (Fukumori et al., 1985; Brown-Peterson and Salin, 1993), and was not induced by environmental stressors including H_2_O_2_ and intense light (Long and Salin, 2000). Additionally, a catalase-peroxidase enzyme was purified from *H. marismortui* (Cendrin et al., 1994).

Superoxide dismutases provide protection from oxidative damage by catalyzing the dismutation of 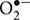 to O_2_ and H_2_O_2_ (Cannio et al., 2000; Imlay, 2003). The yielded H_2_O_2_ is not only less toxic than its 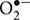 precursor, but also may be subsequently scavenged by hydroperoxidases. The presence of superoxide dismutase has been verified in *H. salinarum* (May and Dennis, 1987) and *H. volcanii* (May et al., 1989). In *H. salinarum*, the encoding gene (*sod*) is positioned adjacent to that of photolyase (Takao et al., 1990). Superoxide dismutase activity has been shown to increase in response to elevated intracellular 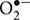 in the aforementioned organisms (May and Dennis, 1989; May et al., 1989; Brown-Peterson and Salin, 1993); however, activity in *H. salinarum* decreased with prolonged exposure, yet was sustained in *H. volcanii*.

The superoxide dismutase of *H. salinarum* is associated with cofactor Mn(II), as opposed to Fe(II) (May and Dennis, 1987; May et al., 1989). It has been shown that *H. salinarum*, as well as the highly radioresistant model bacterium *Deinococcus radiodurans*, have higher intracellular ratios of Mn to Fe than less radiation-resistant organisms (Daly et al., 2004; Kish et al., 2009). While intracellular Mn does not directly provide protection against DNA damage in *H. salinarum* (Robinson et al., 2011), it is hypothesized to play a role in protecting DNA repair proteins from oxidative damage via antioxidant activity (Daly et al., 2007). Indeed, Mn complexes (with phosphates and small organic molecules) have been shown to reduce protein carbonylation (Daly et al., 2010; Matallana-Surget and Wattiez, 2013), a recognized consequence of UV-induced oxidative stress, in *H. salinarum* (Robinson et al., 2011).

One strategy for maintaining osmotic balance with the extracellular environment employed by certain groups of halophilic archaea is to accumulate ions intracellularly, particularly K^+^ and Cl^-^ (da Costa et al., 1998; Oren et al., 2002; Oren, 2008). Concentrated Cl^-^ attenuates oxidative damage by transferring an electron to •OH, producing a hydroxyl anion and atomic chlorine (Cl •) (Shahmohammadi et al., 1998). The subsequent reaction of Cl • with Cl^-^ produces chloride radicals (Cl_2_^∙-^), which are less damaging to DNA than •OH (Ward and Kuo, 1968). Cl^-^ and Br^-^ have been shown to protect DNA from oxidative damage incurred by γ-radiation (Shahmohammadi et al., 1998; Asgarani et al., 1999; Daly et al., 2004; Kish et al., 2009). Kish et al. (2009) further demonstrated that *H. salinarum* accumulates fewer base oxidation products than the non-halophilic *D. radiodurans* when subjected to the same doses of γ-radiation. Potassium chloride also suppresses the formation of CPDs in *H. salinarum*, although it appears to play a larger role in protecting from γ-radiation (Asgarani et al., 1999).

Other common pathways for oxidative damage avoidance, such as thioredoxin/glutaredoxin systems and peroxiredoxins, have been observed in archaea, particularly methanogens (Pedone et al., 2004; Erkel et al., 2006), but remain poorly described for halophilic archaea. However, the presence of γ-glutamylcysteine, a known detoxifier of H_2_O_2_ and 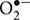 (Quintana-Cabrera et al., 2012), has been observed in millimolar concentrations in *H. salinarum*, *H. volcanii*, *H. marismortui*, and *Halorubrum saccharovorum* (Newton and Javor, 1985; Sundquist and Fahey, 1989).

Altogether, *H. salinarum* demonstrates a remarkable capacity to withstand H_2_O_2_ and 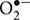. Kaur et al. (2010) observed fairly constant cell survival after 2 h of exogenous H_2_O_2_ exposure up to a threshold of approximately 30 mM H_2_O_2_, after which small increases in concentration induced significant loss. A similar effect was observed on cell growth. For comparison, cell survival of *E. coli* reached 10% after 20 min of exposure to 20 mM H_2_O_2_ (Asad et al., 1998). *H. salinarum* cell survival and growth in the face of 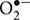 decreases more gradually, with 20–30% loss of survival occurring at approximately 4 mM paraquat, a compound that generates 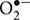 during metabolism (Kaur et al., 2010). It is difficult to compare studies of paraquat toxicity among these microorganisms due to its sensitivity to growth conditions, especially NaCl concentration (Kitzler and Fridovich, 1986). Nevertheless, Korbashi et al. (1986) observed 90% cell loss of *E. coli* treated with 0.75 mM paraquat for 30 min, and Kitzler et al. (1990) observed significant loss after 2–4 h exposure to 2.5 mM.

### Polyploidy

DNA damage, if unrepaired and replicated, can lead to mutation. This underscores the paradigm that while intact DNA is critical to survival, mutation is critical to evolution (Friedberg, 2003). Much has been written about duplication of genes as an evolutionary strategy, since one functional copy allows other copies to change DNA sequence over time (reviewed in Zhang, 2003). However, little has been discussed about the use of polyploidy as a strategy for genome protection. In the case of halophilic archaea, which inhabit UV-intense, hypersaline environments, one mechanism for photoprotection might be simply gene duplication, or in this case, genome duplication.

Halophilic archaea have more than one copy of their genome, and some species have up to 25 copies during their fastest growth phase (Breuert et al., 2006). This polyploidy may provide redundancy of genetic information and can lead to gene conversion or back mutation (Soppa, 2011). Gene duplication has notably led to a variety of rhodopsins in archaea (Ihara et al., 1999). In addition to evolutionary potential, polyploidy provides a nutritional phosphate storage mechanism (Zerulla et al., 2014). With respect to photoprotection, polyploidy would give halophilic archaea more resistance to DNA damaging conditions (Kottemann et al., 2005; Soppa, 2011; Zerulla et al., 2014) such as UV-exposure or desiccation. Logically, increasing the number of copies of a given gene should reduce the probability of its function being lost to DNA damage globally.

A relatively slow rate of global genome repair of CPDs has been reported in polyploid halophilic archaea *H. salinarum* and *H. volcanii* by comparison to the monoploid archaeon *Sulfolobus solfataricus* (Dorazi et al., 2007; Romano et al., 2007; Stantial et al., 2016). Stantial et al. (2016) proposed that this may be attributed to the larger amount of DNA that must be scanned and repaired in polyploid organisms, suggesting a potential tradeoff to the advantage of genome duplication. Also, it should be noted that in yeast, polyploid (4–10 genome copies) cells show no advantage over diploid cells in resistance to ionizing radiation (Mortimer, 1958; Mable and Otto, 2001). To date, there are no UV survival studies probing the significance of ploidy in halophilic archaea.

### Genome Composition

**FIGURE 5:**
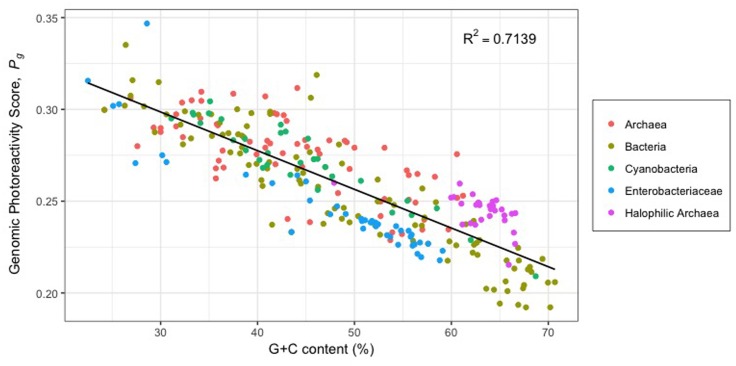
Theoretical genomic photoreactivity based on bipyrimidine signature (*P*_g_) vs. G+C content (%) of 29 halophilic archaea and 243 other prokaryotic genomes (adapted from Jones and Baxter, 2016). *P*_g_ is calculated as the weighted sum of a genome’s bipyrimidine incidences: *P*_g_ = 1.73(TC_i_) + 1.19(TT_i_) + 0.61(CT_i_) + 0.39(CC_i_). Bipyrimidine incidence corresponds to bipyrimidine frequency divided by genome size. Weighting coefficients represent the intrinsic photoreactivity of each bipyrimidine sequence, determined experimentally by Matallana-Surget et al. (2008) as the ratio between the frequency of photoproducts (CPDs and (6-4)PPs) and bipyrimidine incidences in DNA with varying G+C content.

Direct UV damage to DNA predominantly occurs through the cyclization of adjacent pyrimidine nucleotides, producing CPDs, or by the formation of covalent bonds that produce 6-4PPs (**Figure 2**) (Besaratinia et al., 2011). The photochemical susceptibility to lesion formation differs among the four bipyrimidine sequences, decreasing in the order of (5′ to 3′) TC > TT > CT > CC (Matallana-Surget et al., 2008). The more photoreactive bipyrimidines being T-containing, it has long been suggested that organisms with high G+C content, such as halophilic archaea (63.1% G+C on average) (Jones and Baxter, 2016), may be less susceptible to UV-photodamage (Haynes, 1964; Setlow and Carrier, 1966; Joux et al., 1999; Kennedy et al., 2001). Indeed, high G+C content is correlated with a photoprotective bipyrimidine signature (**Figure 5**) (Jones and Baxter, 2016).

While halophilic archaea do have lower genomic photoreactivity with respect to bipyrimidine signature (*P_g_*) than most other microorganisms, it should be noted that they have higher *P*_g_ scores than others with comparable G+C content (**Figure 5**). Interestingly, halophilic archaea have significantly higher incidences of 5′-TC-3′ sites than the average bacterium, archaeon, or random DNA sequence of comparable G+C content (Jones and Baxter, 2016). It has been proposed that this feature is attributed to a demand for acidic amino acids (Zhou et al., 2007), an important adaptation to protein function in high salinity (Kennedy et al., 2001). Notwithstanding the high incidence of 5′-TC-3′ sequences in halophilic archaea genomes does increase susceptibility to bipyrimidine lesion formation, there is, paradoxically, a photoprotective benefit to such: the associated amino acid bias equips these microorganisms with fewer residues susceptible to ROS (Zhou et al., 2007).

The high G+C content of halophilic archaea also decreases their susceptibility to photooxidative DNA damage. Wei et al. (1998) observed a negative relationship between G+C content and the formation of 8-hydroxy-2′-deoxyguanosine (8-OHdG), a guanine oxidation product, in UV-irradiated DNA. These authors hypothesized that thymidine may serve as an intrinsic photosensitizer and therefore, its limitation reduces ^1^O_2_ generation.

## Conclusion and Insights

DNA damage by UV radiation is repaired by all life on Earth. This commonality suggests that our last universal common ancestor (LUCA) had DNA repair systems in place that allowed life to proceed in the presence of high solar irradiance. This was especially important for phototrophic and photosynthetic organisms, which derive energy from light. Early aquatic life was likely exposed to an influx of intense short-wavelength UV as the Earth had no ozone layer (Cockell, 1998; Bérces et al., 2006; Westall et al., 2006). As evolution proceeded, all three domains of life retained the machinery to fix CPDs, (6-4)PPs, DSBs, SSBs, and other oxidative damage. However, archaea possess repair genes that are homologous with both eukaryotes and bacteria, indicating an accumulation of DNA repair strategies that go beyond LUCA’s required set of genes (DiRuggiero et al., 1999; Eisen and Hanawalt, 1999; White, 2003). Thus, the phototrophic halophilic archaea would certainly be expected to have particularly robust repair systems that manage their intense UV exposure and other challenging environmental conditions such as osmotic stress, low water activity, and desiccation.

For all organisms examined, UV-induced DNA damage and repair has been oversimplified in the literature, leading to misperceptions that the primary concern is the accumulation of CPDs and the primary means of handling those is through PHR or NER. Also, it is common to see references to TT as the most significant lesion of concern (e.g., Goo et al., 2004), when in fact each bipyrimidine sequence is susceptible to UV-induced bond rearrangement, and 5′-TC-3′ is more photoreactive than TT (Matallana-Surget et al., 2008).

**FIGURE 6:**
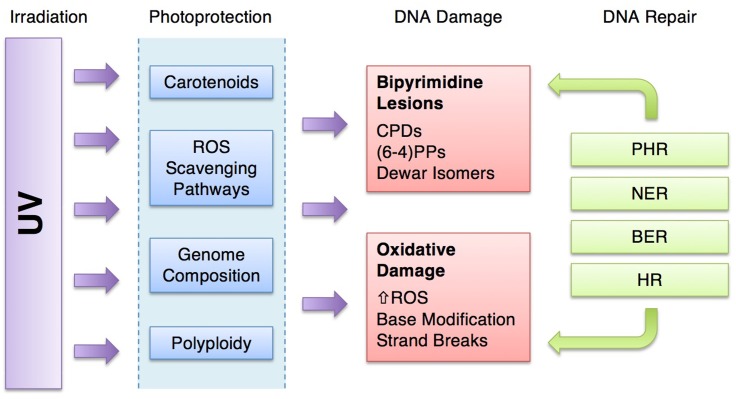
Ultraviolet-resistance strategies of halophilic archaea. UV irradiation is attenuated by photoprotective mechanisms, lessening the damage to DNA. The damage that does result may be repaired by a suite of DNA repair systems. [UV, ultraviolet radiation; ROS, reactive oxygen species; CPDs, cyclobutane pyrimidine dimers; (6-4)PP, pyrimidine (6-4) pyrimidone photoproducts; PHR, photoreactivation; NER, nucleotide excision repair; BER, base excision repair; HR, homologous recombination].

In this review, we have attempted to broaden our understanding of the complexity of types of damage, in particular understanding the impact of UV irradiation on the formation of ROS (**Figure 3**). Likewise, we have been inclusive of the repair systems that address the various types of damage. HR, for example, is often left out of discussions of the repair of UV damage when in fact, *radA*/*recA*/*rad51* mutants are UV sensitive, pointing to the significance of this system. It is critical to understand that there are multiple and overlapping repair pathways for specific types of damage (**Table 1**) (Eisen and Hanawalt, 1999). Indeed, the fate of a (6-4)PP lesion may be repair by either PHR, NER, or BER. If the (6-4)PP is not repaired, it can cause a replication fork to stall, which will activate HR (**Figure 6**).

Halophilic archaea have all of these repair systems, but they also are adept at damage avoidance, preventing the effects of high UV doses on their cellular molecules in the first place. For microorganisms under UV assault, photoprotection provides some energetic advantage by reducing the demand for repair machinery (**Figure 6**). Halophilic archaea possess a unique combination of photoprotective mechanisms including pigmentation, ROS scavenging, genome signatures and polyploidy. These attenuate damage and reduce the burden on the repair systems.

The coloration of halophilic archaea and their environments (**Figure 1**) suggests a link between pigments and photoprotection, prompting early research on carotenoids and UV exposure (e.g., Dundas and Larsen, 1963). While a number of studies provide evidence that carotenoids are essential to UV-resistance in halophilic archaea, none clearly resolve the connection between UV damage and photoprotection by carotenoids since these pigments absorb in the visible light spectra and not in the UV (e.g., Lichtenthaler and Buschmann, 2001). This makes it difficult to explain the observations that carotenoids in halophilic archaea prevent CPD lesions (Baxter et al., 2007) and provide for a more robust PHR system (Sharma et al., 1984). Also, UV screening compounds are typically aromatic, such as melanin in animals, and most carotenoids are not (Cockell and Knowland, 1999). In a direct experiment, no passive UV screening was detected in pigmented vs. non-pigmented *Halobacterium* strains (Sharma et al., 1984). One role for carotenoids in indirect photoprotection from UV light is clear: as antioxidants, they function in protecting from oxidative damage (Krinsky, 1979; Truscott, 1990; Miller et al., 1996; Saito et al., 1997; Young and Lowe, 2001; Stahl and Sies, 2003; Mandelli et al., 2012; Igielska-Kalwat et al., 2015; Islamian and Mehrali, 2015). It is also possible, that instead of direct absorption, they act as secondary pigments as in plants, to dissipate excess excitation energy (Young, 1991), thereby protecting light gathering proteins such as bacteriorhodopsin, which absorbs light in visible light wavelengths similar to carotenoids (Oesterhelt and Stoeckenius, 1971; Stoeckenius and Lozier, 1974).

Ultraviolet radiation facilitates oxidative damage by generating ROS (**Figure 3**) (Cadet et al., 2001; Kawanishi and Hiraku, 2001; Cadet et al., 2005), which in halophilic archaea are detoxified by a number of overlapping systems beyond carotenoids. Carotenoids effectively scavenge •OH and ^1^O_2_ (Truscott, 1990; Saito et al., 1997; Young and Lowe, 2001; Stahl and Sies, 2003; Igielska-Kalwat et al., 2015; Islamian and Mehrali, 2015), while hydroperoxidases, superoxide dismutases, and γ-glutamylcysteine, work together to scavenge 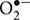 and H_2_O_2_ (Cannio et al., 2000; Imlay, 2003; Pedone et al., 2004). Intracellular ions have also demonstrated the capacity to attenuate oxidative stress (Shahmohammadi et al., 1998; Asgarani et al., 1999; Daly et al., 2004; Kish et al., 2009), although this mechanism remains poorly studied with respect to UV radiation. Altogether, the ROS quenching afforded by these systems protects halophilic archaea from UV radiation not only by preventing oxidative DNA damage, but also through preserving the integrity of DNA repair systems and other enzymes, membranes, metabolic pathways, and a number of other cellular components sensitive to oxidative stress.

Halophilic archaea are distinguished by genomic signatures, namely, high G+C content, low TT bipyrimidine incidence, but high 5′-TC′-3′ incidence (Jones and Baxter, 2016). The literature suggests that these features should confer some UV resistance through limiting photoreactive sequences and oxidative damage (Wei et al., 1998; Zhou et al., 2007; Jones and Baxter, 2016), although the question remains: how much resistance overall? UV-irradiation experiments evaluating the LD_50_ of *Pseudomonas aeruginosa*, a bacterium with similar G+C content to halophilic archaea, suggest other strategies (DNA repair efficiency, pigmentation) confer more photoprotection than genomic signatures (Baxter et al., 2007). Furthermore, halophilic archaeon *H. walsbyi* has a G+C content of only 47.9% (Bolhuis et al., 2006), yet still thrives in the same environment as, for example, *H. salinarum* (65.9% G+C) (DasSarma et al., 2001). Indeed, as early as 1964, Haynes noted that the UV-sensitivity of microorganisms was not correlated with thymine frequency in the genome (Haynes, 1964).

Desiccation of hypersaline environments is a natural cyclic condition (Mancinelli et al., 2004; Baxter et al., 2007), and when their environment dries up, halophilic archaea are trapped in fluid inclusions inside salt crystals and are capable of surviving desiccation over geologic time scales (reviewed in Lowenstein et al., 2011). When embedded in salt, they are particularly resistant to UV light (Fendrihan et al., 2009). The authors of this study attribute the UV-resistance of the three tested species to the properties of halite, which have color centers that could attenuate the UV radiation. This may result in absorption of UV light and re-emission at longer wavelengths. This environmental UV screening from minerals in the environment is considered a passive approach to photoprotection (Cockell and Knowland, 1999), such as the formation of colonies or biofilms (Gao and Garcia-Pichel, 2011). Polyploidy in halophilic archaea affords an obvious potential benefit in surviving UV-irradiation in that genes are duplicated and thus intact copies are readily available (Zerulla and Soppa, 2013). But polyploidy may also be a strategy for surviving long-term desiccation. Even over geologic time in salt crystals, DNA can be preserved since polyploid cells can build intact chromosomes from DNA fragments (Kottemann et al., 2005). In fact, three species recovered from an Eocene salt formation exhibited an average genome copy number of 6–8 (Jaakkola et al., 2014). Thus, polyploidy may be a strategy that is aids in DNA protection is a variety of ways.

Other lifestyle considerations may impact both the UV exposure and photoprotection of halophilic archaea. Phototaxis, for example, exposes cells to more sunlight and thus a higher UV dose. Halophilic archaea use gas vacuoles to move in the water column (Simon, 1978), which allows for efficient light-gathering from bacteriorhodopsin (Blaurock and Stoeckenius, 1971; Oesterhelt and Stoeckenius, 1971; Stoeckenius and Lozier, 1974; DasSarma et al., 2001; Lanyi, 2004). Interestingly, gas vacuole genes are downregulated after UV exposure, suggesting a method to move away from the radiation source (Baliga et al., 2004). Also, there appears to be no passive shielding from the gas vacuoles; Simon (1980) found that *H. salinarum* defective in gas vacuole production had no significant sensitivity to UV exposure.

Halophilic archaea have evolved in the presence of high sunlight exposure. They have accumulated an arsenal of photoprotective strategies to accompany their DNA repair machinery (**Figure 6**). Which of these is the most critical to survival? Mutant studies help us tease apart the critical pieces, but comparatively, it is hard to distinguish one beneficial strategy from another. And of course, it may depend on environmental conditions or a host of other factors. For example, the biology of halophilic archaea may be suspended if they are metabolically dormant in salt crystals, but chemistry could still occur. In particular, over geologic time scales, this points to ROS scavenging as paramount for survival over time. Contrary to this, in a warm summer climate, halophilic archaea in their logarithmic growth phase may depend on a combination of carotenoids, polyploidy and genomic signatures to protect DNA and minimize repair efforts. Perhaps, then, it is the dynamic environment and lifestyle of the halophilic archaea that necessitate a suite of approaches to maintaining the integrity of their DNA.

## Author Contributions

DJ and BB contributed equally and made substantial contributions to the intellectual design and the writing and editing of this review article. Both are accountable for all aspects of the work.

## Conflict of Interest Statement

The authors declare that the research was conducted in the absence of any commercial or financial relationships that could be construed as a potential conflict of interest.
